# Activity Performance Priorities for Adults Experiencing Homelessness: Insights from Management and Staff at a Transitional Housing Facility

**DOI:** 10.3390/ijerph22010021

**Published:** 2024-12-28

**Authors:** Bernard Austin Kigunda Muriithi

**Affiliations:** 1 Department of Communication Disorders and Occupational Therapy, College of Education and Health Professions, University of Arkansas, Fayetteville, AR 72701, USA; muriithi@uark.edu; Tel.: +1-479-575-5028; 2 College of Health Professions, University of Arkansas for Medical Sciences (Northwest Arkansas Campus), Fayetteville, AR 72703, USA

**Keywords:** homeless, transitional housing, daily activity, occupational therapy, mental health, physical health, independence, community living, boredom, isolation

## Abstract

For people experiencing homelessness (PEH), the provision of affordable housing has been recognized as the most crucial intervention for improving housing stability and facilitating substance abuse treatment. However, evidence indicates that providing housing does not significantly improve substance abuse, mental health, or physical health outcomes. Optimal participation in essential daily activities has been shown to improve health outcomes and support independent living, but there is limited research that identifies activity performance priorities among PEH living in transitional housing. The present study investigates activity performance priorities using the focus group methodology. Management and staff from a transitional housing facility participated in a focus group discussion (90 min) and a follow-up (member checking) session (75 min), to deliberate on this topic. Emerging priorities for activity performance include activities of daily living [personal hygiene]; instrumental activities of daily living [house maintenance, meal preparation, and transportation]; health management [mental health, physical health, medical appointments]; social participation [building healthy relationships]; work [seeking and/or maintaining]; sleep; and leisure [recreational] activities. Community members and other stakeholders can support PEH in transitional housing by facilitating participation in these activities, and researchers should evaluate the effects of specific activity-focused interventions on health.

## 1. Introduction

Homelessness is a significant public health crisis that seems to be worsening. On a singular night in 2023, approximately 653,100 individuals were identified as experiencing homelessness in the United States [1]. The US Department of Housing and Urban Development reported an increase of 70,650 people experiencing homelessness in 2023 compared to 2022, reflecting a 12% increase. The report also indicated that homelessness in the United States has a disproportionate impact on minority groups. African Americans, although they comprise only 12% of the overall US population, represented 37% of people experiencing homelessness (PEH). Similarly, American Indians, Alaskans, Pacific Islanders, and Native Hawaiians, which together comprise less than 1% of the population of the United States, represented 5% of the homeless demographic.

Interventions aimed at first providing housing have rightly been considered the most fundamental for PEH [2], but evidence shows that Housing First and comparable housing models have not significantly improved substance abuse [3], mental health [3,4], and physical health outcomes [5]. Therefore, more studies are needed to provide evidence for interventions that have a more significant impact on health among PEH.

A possible intervention is to increase participation in meaningful daily activities [6,7,8,9,10]. Evidence has shown that everyday activities influence health, a claim that has been reiterated in the field of occupational therapy since its inception [11,12]. The practice of occupational therapy is based on the belief that everyday activities that people carry out (i.e., occupations) are fundamental to health promotion, wellness, health maintenance, disease prevention, and adaptation [13].

Ordinary everyday activities have been shown to positively influence sense of coherence [14], psychological resilience [15], and overall health [16]. Resilience and sense of coherence are both measurable constructs that have recognized associations with health and well-being. Both constructs are strongly related to participation in activities. Research has shown that interventions focused on enabling activity performance (i.e., physical exercise, sailing, music, cultural activities, relaxation activities, etc.) promote sense of coherence and subsequent health [14]. Activity restrictions have been linked to poor resilience. For example, during COVID-19 lockdowns, people involved in outdoor activities, physical exercise, enough sleep, prayer, or social engagement obtained higher resilience scores [15]. In a separate study, activity performance (sleep, play and recreational activities, work, personal hygiene, and healthy eating) was also identified as a significant predictor of overall health during COVID-19 lockdowns [16]. These studies suggest that the provision of resources and skills for participation in activities could have significant health implications for PEH.

The current study aimed to identify the areas of activity performance in which residents of a transitional housing facility (New Beginnings) have challenges and the need for support. The study sought to answer the following question: What are the activity priorities for PEH at New Beginnings? Answering this question provides stakeholders with the insights necessary to identify potential pathways for collaboration in initiatives aimed at promoting health, fostering independence, and facilitating the transition to independent housing. Community collaborators might provide material resources or volunteer their services to enhance engagement in these activities. Although the activity priorities at this facility may parallel those observed at similar facilities in the United States and elsewhere in the world, this study is not designed to produce generalizable findings.

## 2. Methods

The Institutional Review Board of the University of Arkansas Fayetteville granted expedited approval for this study under Protocol No. 2309492440. Informed consent was obtained from all participants after addressing all inquiries related to the study.

### 2.1. Design

The present study employs a qualitative research design, using focus group methods. Focus groups are characterized by six attributes: the presence of a moderator, an observer, a small group of participants capable of offering research-specific insights, a defined subject or theme, structured rather than spontaneous discussion, and unconventional data collection methods [17]. The advantages of focus groups include the capacity to facilitate an extensive array of information by fostering synergies among participants, the cascading effect of thoughts and ideas, stimulation, serendipity, spontaneity, salience, and specialization [17]. This approach was deemed appropriate as the study aimed to elicit ideas from participants based on the diverse roles they held within the transitional housing facility. Participants were expected to provide different insights based on their respective roles.

The study design was intended to serve an exploratory–descriptive function, considered suitable for researchers seeking new knowledge that may not have been previously uncovered [18]. The exploratory–descriptive function prioritizes breadth and heterogeneity of information over depth, intentionally eliciting varied information from participants who have expertise on the subject matter. The exploratory–descriptive function is descriptive rather than analytical, exploratory rather than investigative [17]. The study was not designed to address other functions of focus groups, including in-depth analysis [deepening perspectives], experiments [to provide a structured explanation of outcomes in an experiment], and evaluation [to provide arguments for or against products, policies, and programs] [17].

### 2.2. Sampling

The individuals invited to participate in the study are recognized as experts in the subject matter, due to years of experiencing daily interactions with PEH in various capacities. Focus groups leverage the knowledge of experts, the individuals possessing experience and a deep understanding of the phenomenon under investigation. The study sample comprised individuals performing various roles as management and staff personnel at New Beginnings. This was intended to ensure that diverse perspectives were presented based on the roles of participants. For example, the program manager focused on securing grant funding and formulating collaborative initiatives with other community stakeholders, while the director of healthcare facilitated residents’ access to health insurance and essential healthcare services. The roles performed by staff at this facility include a housing specialist, director of community engagement, case manager, support specialist, garden coach, and financial administrator. Given the extensive experience of the participants in their respective roles and duties, the group was considered heterogeneous and highly knowledgeable about the subject under investigation.

Five participants attended the meetings (i.e., the Program Director, Housing Specialist, Director of Community Engagement, Case Manager, and Director of Healthcare). Considering the Information Power (IP) model [19], a sample of five highly knowledgeable participants can be considered adequate in a qualitative study. According to the Information Power model, the study objectives are regarded as narrow [concentrating on activity priorities], the sample specificity is deemed dense [participants had diverse knowledge or expertise determined by their roles in the facility], homelessness is regarded as generally well theorized [there are many relevant theories to explain this phenomenon], the quality of is dialogue strong [participants and researcher have rather deep conversations], there is high sample variation [diverse roles represented], and lastly, analysis is focused on just one case facility and does not aim for generalizable results. Given these considerations, the selected sample provided extensive data that are varied and rich in response to the study question.

### 2.3. Data Collection

**Figure 1:**
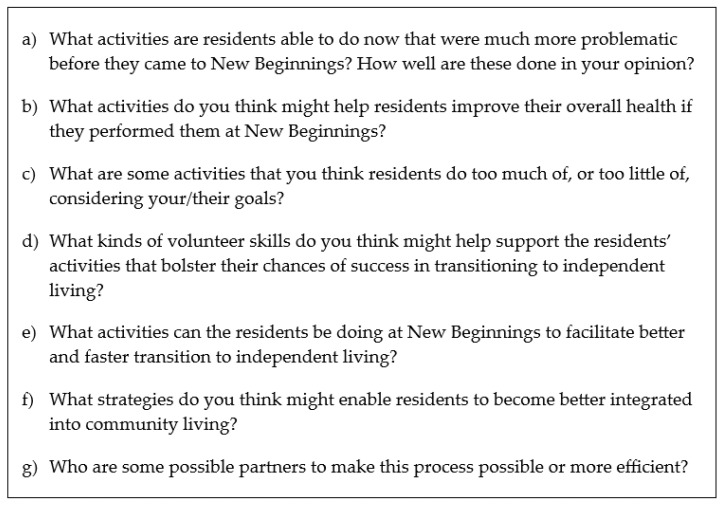
Discussion questions.

Data were collected in one focus group meeting lasting 90 min, with a follow-up 75 min session for member checking. The session was recorded and transcribed. In the first session, the two investigators alternated between the moderator and observer roles, using the questions in Figure 1 as a guide, with emerging issues leading to additional probing questions. The moderator kept the group focused on activity priorities while seeking perspectives from all participants, as the observer took notes. The second session had only one investigator present, who served as moderator. At the time of the second meeting, analysis had been conducted to identify the activity priorities of the first focus group meeting. Consequently, the second session was dedicated to member checking, with the objective of validating the analysis results and providing additional depth or clarifications where necessary.

### 2.4. Analysis

The data analysis process involved the identification of relevant themes within the data, the highlighting of activities as they were referenced in the discussions, and the delineation of associated concerns. Six groups of occupational therapy doctoral students separately conducted comprehensive analyses of the data. They meticulously identified activities from the discussion themes and outlined what they considered priorities in activity performance and related issues. The summary of findings from these analyses is presented in Appendix A. The integration and abstraction of the themes was carried out by the primary investigator, who also considered observational notes taken during the focus group meeting. Subsequently, the principal investigator reconvened a second follow-up session to achieve participant consensus on the results and to enrich the analysis where necessary. Lastly, a draft of this manuscript was sent to the focus group participants to ask for feedback and recommendations prior to finalizing the manuscript.

## 3. Results

New Beginnings and similar facilities can derive substantial benefits from collaborations with partners who possess the ability and willingness to support skill development and participation in the enumerated activities. The activity priorities at New Beginnings include maintaining personal hygiene, maintaining cabins/house cleaning, cooking, managing transportation, managing mental health symptoms, scheduling and keeping medical appointments, physical activity, building healthy social relationships, activities to promote mental health, scheduling, and keeping medical appointments, increasing physical activity, social interaction, employment, sleep, and leisure activities.

### 3.1. Maintaining Personal Hygiene

Personal hygiene was identified as an activity priority area. Residents formerly lived on the streets, where facilities for engaging in activities such as bathing, toileting, and grooming were difficult to access. Residents were frequently observed to neglect personal hygiene despite New Beginnings providing the necessary resources to facilitate these activities. It was stated that “everyone [residents] struggles with all these things [hygiene]” (Speaker 3). Prior to coming to New Beginnings “the [their] living environment is [was] full of feces and urine, diseases, old moldy clothes, tents upon tents upon tents, old possessions, wet possessions and other people’s possessions” (Speaker 3).

Given this background, it was understandable that staff needed to intervene to assist residents with daily routine tasks such as showering and undertaking oral hygiene activities regularly. Given that residents are expected to transition to independent living when direct supervision of this activity is absent, it is imperative that they develop the ability to perform personal hygiene more autonomously.

### 3.2. House Maintenance

Residents had difficulties in sustaining a clean and organized living environment, as suggested by the observation that “this is why the cabins are filthy” (Speaker 6). To clean up living spaces, remove trash, and organize their rooms, staff encouragement and support was needed: “Staff reinforcement with people … the tools for managing [their] own time and having meaningful activities [done] every day” (Speaker 3). The transition to independent living within the community requires improved autonomy in house maintenance; otherwise, residents are likely to face substantial challenges with independent living in the community.

### 3.3. Meal Preparation

Participants suggested that acquiring the skills necessary to prepare nutritious meals is essential to ensure that residents have access to healthy food upon leaving New Beginnings. At the facility, residents were not involved in meal preparation due to concerns about their competence and safety in handling food. The facility relies on volunteers for meal preparation. However, it was recognized that building cooking skills would facilitate the transition to independent living, as it would allow residents to afford nutritious meals by preparing them independently rather than dining in restaurants. Inadequate meal preparation skills were part of the reason why a participant remarked: “I will frame it like this… I think the need for skill development is so pronounced in every single person here” (Speaker 3). It was suggested that one-on-one support can be of help: “I think one-on-one time with each resident individually would help them to reach their goals, their housing goals” (Speaker 2).

### 3.4. Managing Transportation

Participants suggested that the inability to navigate the community substantially impedes the ability of residents at New Beginnings to live independently within the community. It limits access to shopping centers, medical facilities, and recreational activities. Transportation posed significant challenges because the facility is located in a part of the city where access to reliable public transportation is problematic. The participants felt that the residents had the ability to use public transportation, but the location of this facility made access difficult. Consequently, this issue appears to be predominantly attributable to environmental factors rather than deficiencies in the residents’ performance capabilities, as indicated: “I think most people know how to do public transit…for what it is [unreliable] there are certain destinations that are hard to access…[including] the Community Clinic” (Speaker 3).

### 3.5. Managing Mental Health Symptoms

For residents to thrive and live autonomously after their transition from New Beginnings, participants felt it is vital that they independently engage in activities that help them manage their mental health, a very common barrier to progress: “mental health is the biggest common denominator…I’d say the biggest need” (Speaker 6). Mental health issues were quite prevalent among the residents. The mental health symptoms often escalated into emergencies. It was stated that:

“I think working in mental health, it kind of catches my eye a little quicker…people that are in a crisis need more attention at the time of the crisis and not having that available here, it puts a damper on it [chances of success]” (Speaker 6)

The necessity for access to a mental health specialist often emerges at times when the availability of a professional is least probable:

“They do need a professional, and we cannot get them there [an immediate appointment with a specialist]. We can’t because they [professionals] are not accessible…. They say well, come on Tuesday at 7:30 in the morning” (Speaker 6).

Substance use and drug addiction were also common, and they are known to aggravate mental health symptoms. It was suggested that substance use was the root cause of behavioral problems:

“Alcoholism and drug addiction is [are] also at the root of a lot of behavior issues that are really challenging to support people with, and …it may be… they didn’t handle a conflict well…but the root of it was their dependence on the substance” (Speaker 3).

The identification of specific activities that would enable residents to independently manage their mental health symptoms is considered essential to promoting health and independence.

### 3.6. Managing Medical Appointments

Given that many residents at New Beginnings have medical conditions, participants noted that consistent medical care is needed. It is therefore a matter of concern that many require assistance in arranging and adhering to appointments with healthcare providers. In many cases, staff have assumed this responsibility; otherwise, the task of scheduling or attending appointments is frequently not followed as expected. Participants saw a need for residents to build the capability to independently arrange and attend appointments with healthcare providers in order to maintain their health and receive the necessary medical attention.

“[Managing appointments] is a goal that we work towards because when they move out and you think about them moving out, then they ultimately need to be able to take care of those things on their own or we need to assess if they’re able to take care of those things on their own or if it’s part of the permanent supportive housing…The reason that we need permanent supportive housing is that some people will never be able to do that” (Speaker 6).

### 3.7. Managing Physical Activity

After stating that residents had extended periods of living on the streets, participants felt that the residents at New Beginning now experience improved nutritional intake, which starkly contrasts with their dietary habits prior to their arrival at the facility. However, a new issue was beginning to manifest itself, which is the increase in body weight. It was stated:

“They eat very well here, but they gain a lot of gain weight…they all complain of gaining weight. You can see they don’t exercise as much” (Speaker 5).

Although residents have options for various physical activities (such as exercise, walking, dog walking, hiking, and gardening on their campus), they do not participate in these activities with sufficient regularity to maintain healthy activity levels and mitigate the risk of obesity and related health conditions.

### 3.8. Building Healthy Social Relationships

Participants noted that the development of healthy social relationships is crucial for the residents at New Beginnings. Despite residing within a community, participants indicated that the majority of the residents experienced feelings of loneliness and isolation. Upon arriving at New Beginnings, residents do not have the autonomy to choose their neighbors. Certain individuals expressed a sense of loss regarding friends who were left behind on the streets, while others found it challenging to engage socially with other urban residents due to feelings of stigmatization, which in turn intensified their tendencies toward self-isolation. The participants perceived that residents experienced negative attention when they were in the community: “It’s really difficult to get them out of their shell, out of their comfort in their space” (Speaker 2). Speaker 3 added:

“I do think places [outside facility] feel like… scary…they feel like [they] stand out, don’t wanna go as a group because… [they] don’t want to be with the other homeless that would be part of your homeless day trip to the museum…I think there’s a little bit of that stigma” (Speaker 3).

Interventions can help residents improve social interaction skills, so that residents can feel more comfortable interacting with other people, but getting residents motivated enough to participate has been challenging.

Participants ascribed the tendencies toward aggression to probable experiences of violence and trauma encountered on the streets. It was proposed that assisting the residents could be achieved through role models who may facilitate engagement in normal social interactions. Participants explained the potential role of volunteer role models, positing that residents could benefit from individuals capable of modeling:

“We do have opportunities daily for that kind of normalizing social interactions [through modeling]. And I’ve also said to volunteers. they normalize proper social interactions and normalize good behavioring and that has really helped a lot” (Speaker 7).

A participant articulated their perception of what might occur in the cognitive processes of certain residents who exhibit aggressive tendencies:

“My guard is up …I have to be tough. I have to be strong. I have to be assertive…So I’m going to overdo it, I overdo the aggression to protect myself… overdo it to protect themselves because there is nothing to protect them except their reputation” (Speaker 3).

The mental health counselor described some of the strategies they have tried:

“We also have Mental Health Minutes at the beginning of Thursday meetings, and that’s about when we talk about…maybe if somebody’s yelling at you or you’re upset, take a breath or just walk away, take a walk up and down the road. So, every Thursday we have a step of what somebody could do, not for that particular thing, but any particular mental health minute. And we did have different therapy people involved with us” (Speaker 7).

### 3.9. Seeking and Maintaining Employment

To meet financial obligations (such as rent and other living expenses) after leaving New Beginnings, securing employment was regarded by participants as critical for success. A limited number of residents were employed, and even among those who worked, the job roles exhibited a lack of consistency and sustainability. The development of skills to succeed in a workplace is, therefore, of paramount importance. Additionally, maintaining work requires the ability to manage transportation, seek help and manage health conditions, and sustain motivation for work. Participants reported that interventions associated with work are largely outside their scope of work. After describing one resident who lost a job due to exacerbated mental health symptoms, it was stated:

“To be honest I don’t think we were able to offer support such as job coaching, job retention, skill building …[teaching] how to maintain their job is not something we have the capacity to offer” (Speaker 3).

For those struggling to find jobs, seeking jobs is complicated by criminal backgrounds, necessitating processes that are both costly and difficult to resolve:

“Tenant history, credit history, criminal background [are] barriers. So there the reason that the people maybe not [transition] faster is more due to system barriers around housing and access to housing” (Speaker 2).

The development of daily life skills was identified as significant in stabilizing and preparing residents at New Beginnings for independent living and enhancing health outcomes. However, it was considered insufficient in aiding their transition when housing remains a persistent barrier. Participants, therefore, recognized that New Beginnings needed to collaborate with various stakeholders to effectively address the multifaceted barriers that impede success, including systemic barriers like access to affordable housing, skills for employment tasks, and access to healthcare services.

### 3.10. Maintaining Regular Sleep

At New Beginnings, sleep was often problematic for some residents, with challenges including oversleeping and irregular sleep patterns. Oversleeping was ascribed to the newfound experience of residing in a secure and comfortable environment after prolonged periods of living and sleeping outdoors, where

“They would sleep with one eye open or with shoes on in case they have to get up and run…or did not sleep due to community disturbances and climate changes” (Speaker 6).

New Beginnings offered a secure environment conducive to sleep; however, this led to instances of oversleeping among some individuals, while others exhibited irregular sleep patterns: “now that they are in a safer place that they feel they can sleep, but then they sleep and sleep and sleep” (Speaker 2). Sleep difficulties also existed. It was said:

“I think we’ve improved, but I think it could be better in the way that you know if depression or things like that [can lead to insomnia]. And then people sleeping too much…all day sleep” (Speaker 2). Another participant added:

“But there is [are] disturbances here, so I’ve had people complain about. ‘No, I can’t sleep because…the next door [neighbor] is making these noises or is walking around’” (Speaker 6).

Thus, prioritizing and effectively addressing sleep-related issues at New Beginnings emerges as a potential strategy in fostering health and independence. Addressing sleep problems was viewed as one way to empower residents and unlock their full potential during the day, thereby improving their health and independence.

### 3.11. Engaging in Healthy Leisure and Recreation

New Beginnings had limited capacity to organize recreational activities, but residents responded well when they occurred. Residents struggled to find healthy recreational opportunities. These activities are essential for skill development and to reduce boredom-related issues such as substance abuse and associated problems. Interventions might help, given:

“I do think we’ve all seen like the boredom, the self isolation, the fear of going to a museum where we’ve offered those things. We just have this much that we have the ability to offer to do” (Speaker 3).

Additionally, sedentary lifestyles are common, as suggested: “they watch movies too much. They’re like on their screen all the time” (Speaker 6). Certain organized recreational activity organized by an occupational therapy intern was described thus:

“The activity that was the most engaged in of the OT interns this semester was tie dye. It was by far the best attended; the most joyful noises came from it” (Speaker 3).

In addition, in relation to recreational activities, one participant implied that residents do not appear to be concerned about participating in a variety of activities. They stated:

“I get two meals a day I can definitely live off of… I can get in my cabin and watch my videos all day long and order things from Amazon and nobody cares…I don’t have to get healthier…they have survived they’ve survived for so long; they don’t know that they can thrive” (Speaker 6).

## 4. Discussion

Eleven activity priorities were identified in the analysis, including (1) maintaining personal hygiene; (2) house/cabin maintenance; (3) cooking; (4) managing transportation; (5) activities to manage mental health; (6) managing medical appointments; (7) engaging in physical activity; (8) maintaining healthy social relationships; (9) seeking/maintaining employment; (10) sleep; and (11) healthy leisure/recreation activities. These 11 are potential areas of focus for activity-based interventions that can improve health and independence for New Beginnings residents.

Although personal hygiene is listed as an activity priority area, the underlying problem is not a lack of physical capacity to perform personal hygiene tasks regularly. Rather, it is the result of habits developed when residents lived on the streets, where personal hygiene resources were lacking. This underscores the importance of first providing housing, as reiterated repeatedly in the literature [2,20]. Unsheltered PEH not only lack resources to perform basic daily tasks like personal hygiene, but are more disconnected from healthcare services, have higher rates of substance use and mental health conditions, and are more exposed to violence and trauma risks [20]. Because New Beginnings residents have this type of background, they may not prioritize personal hygiene and therefore often need staff reinforcement to maintain hygiene. Since the goal of residents and the facility is to transition to independent living, participants must improve personal hygiene to prevent negative attention or exacerbate stigma.

Several instrumental activities of daily living (IADLs) emerged as areas of activity priority, including housekeeping, managing transportation, and meal preparation. IADLs support daily life by making it possible for individuals to live independently at home and in a community [21]. Failure to independently perform these activities means that residents always need to be supervised or assisted at home and in the community. Poor house maintenance can result in greater isolation and more stigma, or even eviction from some rental facilities. Transportation management enables people to access shopping centers, healthcare facilities, and other community facilities and resources. Therefore, increasing independence in the performance of these IADLs is understandably a high priority.

Three categories of activity priority relate to health management, defined as “activities related to developing, managing, and maintaining health and wellness routines” [21]. The activity categories include managing mental health symptoms, medical appointments, and physical health. Managing mental health and medical appointments are both key to better health given the very high prevalence of mental health conditions among PEH, which has ranged within 48.4–98% between studies [22,23,24,25]. The statistics agree with the participant’s assertion that mental health conditions are common in this population. Indeed, countless reports have explored the bi-directional relationship between mental health and homelessness, which have highlighted how failed deinstitutionalization policies created potentially bigger issues for PEH with serious mental illness [26]. The need for independence in seeking medical care is further indicated given that up to 70% of PEH reported having physical health problems within the past 12 months [27]. Therefore, the addressing of health and independence among PEH, consistent with the perspectives of study participants, should include strategies that help residents improve the performance of activities classified under health management.

Social interaction activities were another priority area based on the perspectives of the participants, who suggested that residents could benefit from observing and interacting with people who can model good behavior. PEH are commonly stigmatized and dehumanized [28,29,30], and these cognitive and behavioral reactions from society create serious physical and mental health problems [31]. PEH react to society and the various forms of stigma [32] and dehumanization through self-isolation behaviors. Therefore, facilitating activities that nurture positive social interaction and address stigma may be essential to integrate these individuals into the larger community, in addition to creating health-enhancing microcosms in facilities such as New Beginnings. Ideally, facilities such as New Beginnings hope to become a microcosm of society, enabling residents to learn social skills to use in wider society. Activities that promote social interaction and reduce stigma are critical to facilitating success in the performance of many other activities within communities.

Although sleep was reported as an area that improves markedly after residents come to New Beginnings, irregularity in sleep patterns and oversleeping were highlighted as problems with this activity. Good sleep quality is known to restore neural functioning and improve physical and emotional health [33,34], and is associated with better performance in other activities, including work [35,36]. For residents who struggle with healthy sleep patterns, this is an important area of performance that would contribute to overall health, participation in other activities, and the transition to independent living.

Participants also highlighted the importance of work as a priority for residents, given its role as an income generator. A reliable and consistent income source would greatly support the transition to independent living by making housing and other necessary goods and services accessible. In fact, the lack of regular and sustainable employment that pays a living wage is a key contributor to homelessness [37] and a contributor to psychological distress [38]. On the other hand, work was among a handful of activity types associated with better overall health [16]. Therefore, supporting residents to participate in work helps them improve health, in addition to increasing access to resources and fostering independence in community living.

The last priority area identified was leisure activities. These types of activity have hundreds of mechanisms of action through which they influence health [39]. They are known to affect mental health [40,41,42,43], psychological well-being [44], and physical health [45]. Residents in New Beginning have limited opportunities and can benefit from more leisure activities as a way to improve health and well-being. Leisure activities have been identified in studies as helpful in managing mental health symptoms [46,47]. Greater opportunity to participate in leisure activities can help PEH improve health, well-being, social participation, and work-related skills.

This study is limited in that it looks at activity priorities in one facility rather than including perspectives from different transitional housing facilities, which would make the results more generalizable. Furthermore, the exclusion of perspectives from the residents within the facility may overlook substantial insights or potentially divergent opinions concerning this issue.

## 5. Conclusions

The current study identified eleven categories of activity priorities, deriving insights from the management and staff of a transitional housing facility. These 11 categories represent ADLs, IADLs, health management, sleep, work, and leisure activities. Participants see improved performance in these activities as meaningful for the health and independence of residents in this transitional housing. Community collaborators and other entities capable of increasing performance in these daily activities are encouraged to partner with New Beginnings and similar facilities to tackle this complex social and public health challenge. Researchers, by rigorously evaluating the effectiveness of activity-based interventions, could play an essential role in uncovering transformative knowledge to promote health and independence for PEH.

## Data Availability

The raw data supporting the conclusions of this article will be made available by the authors on request.

## Appendix A

This is a table of summarized findings from six groups of occupational therapy doctoral students who conducted analyses in six groups separately.

**Table A1:** Results of six separate analyses.

Group 1 **Social Participation**—Isolation/feeling stigma/not wanting to engage in community. **Community Navigation**—Difficulty accessing/using public transportation **Health management**—Not exercising, not seeking doctor’s appointments **Meal preparation**—Limited skills for cooking **Cabin management**—Unable to maintain clean living space **Substance use problems**—Common recreational activity	Group 2 **Self-care**—Showering, healthy eating, hygiene, sleep **Health management**—Navigating healthcare system, obtaining insurance, attending appointments, exercise **Social engagement**—Finding community, adapting to new environment, building positive relationships **Independent living skills**—Navigating the community, finding sustainable employment, cooking, home management
Group 3 **Mental health management**—Difficulty with emotion regulation, trouble with stress management and decision-making **Daily activity necessities**—Hygiene, sleep, nutrition **Health management**—Mental and physical health neglect, substance use problems, trouble scheduling appointments, arranging transportation, getting insurance. **Community engagement**—Transportation options are limited	Group 4 **Daily living skills**—Need skills in budgeting, grocery shopping and cooking **Health management**—Trouble with transportation, keeping appointments, getting insurance **Community Participation**—Limited or no work, leisure and social activity, problem with transportation **Social participation**—Refraining from social engagement **Excessive sleeping**—Lack of balanced lifestyle
Group 5 **Daily life skills**—Managing medical appointments, budgeting, securing jobs, cooking **Health management**—Exercise, personal hygiene, sleep, dieting, must be reinforced to clean cabins **Community engagement**—Difficulty managing public transportation **Social Participation**—Trouble forming relationships, poor communication methods, unable to resolve conflicts well **Leisure**—Choosing self-isolating activities, watching TV all day	Group 6 **Life skills**—Self-care, money and home management, cooking, planning independently **Health and wellness**—Maintaining appointments with providers **Maintaining a job**—Doing only short-term jobs, need coaching and more skills **Difficulty spending time with others**—Showing aggressive behaviors, feeling stigmatized **Doing enjoyable things**—Drinking and substance abuse, unhealthy recreational activities.
